# Genomic epidemiology of Group B *Streptococcus* colonization among pregnant women in Japan, 2021–2023

**DOI:** 10.1371/journal.pone.0354799

**Published:** 2026-07-30

**Authors:** Chizu Nishi, Hirofumi Ohtaki, Masaki Karino, Hiromi Uekita, Ayumi Sano, Tetsuya Kobayashi, Susumu Kaino, Yoshiki Sakamoto, Satoshi Nakano

**Affiliations:** 1 Department of Clinical Laboratory, Mimihara General Hospital, Sakai, Osaka, Japan; 2 Department of Clinical Laboratory Science, Kansai University of Health Sciences, Sennan-gun, Osaka, Japan; 3 Department of Obstetrics and Gynecology, Mimihara General Hospital, Sakai, Osaka, Japan; 4 Antimicrobial Resistance Research Center, National Institute of Infectious Diseases, Japan Institute for Health Security, Higashimurayama-shi, Tokyo, Japan; Defense Threat Reduction Agency, UNITED STATES OF AMERICA

## Abstract

Group B *Streptococcus* (GBS) colonization in pregnant women is a major risk factor for neonatal diseases; however, molecular epidemiological studies on GBS in Japan remain limited. We characterized 219 GBS isolates recovered from vaginal screening cultures at a general hospital in Osaka Prefecture, Japan between 2021 and 2023. Capsular type, clonal complex/sequence type (CC/ST), virulence factors (pilus islands, Alp family, Srr1/Srr2, and HvgA), and antimicrobial resistance determinants were analyzed using whole-genome sequencing. The predominant lineages were CC19 (27.9%), CC23 (21.9%), and CC1 (21.0%), whereas the hypervirulent CC17 lineage accounted for only 5.5% of isolates. Capsular type Ia, III, and V comprised >60% of the isolates. PI-1/PI-2a (58.0%) was the predominant pilus profile, and *rib* (35.2%) was the most frequent Alp family gene. The hypervirulence-associated markers *hvgA* and *srr2* were confined to capsular types III/CC17 and IV/CC452. All isolates were susceptible to β-lactams and vancomycin, whereas resistance to macrolides, clindamycin, and levofloxacin was observed at varying frequencies. These findings provide a pre-vaccine molecular baseline for maternal GBS carriage in Japan and highlight *srr2*-positive CC452 as a potential emerging lineage of concern.

## 1. Introduction

Group B *Streptococcus* (GBS; *Streptococcus agalactiae*) colonizes the gastrointestinal and genital tracts of approximately one in five pregnant women and is a leading cause of neonatal sepsis and meningitis worldwide [1,2]. Neonatal GBS disease is classified by age of onset into early-onset disease (EOD, 0–6 days), late-onset disease (LOD, 7–89 days), and very-late-onset disease (≥90 days) [3]. Intrapartum antibiotic prophylaxis (IAP), implemented on the basis of antenatal screening, substantially reduces EOD by interrupting vertical transmission during labor. However, IAP does not prevent LOD, which typically arises through various postnatal routes of infection [1].

Maternal vaccination is being pursued to close this prevention gap. Hexavalent capsular polysaccharide–conjugate vaccines (GBS6) targeting globally dominant capsular types have shown favorable immunogenicity in phase 1/2 studies in non-pregnant adults and phase 2 studies in pregnant women [4,5]. In addition, protein-based vaccines targeting conserved alpha-like protein (Alp) family surface proteins (e.g., MinervaX GBS-NN/NN2) have progressed through phase 1 trials in healthy adult women [6,7].

Pre-licensure molecular epidemiological studies are essential for anticipating the vaccine impact and detecting potential capsular types or lineage replacements after vaccine introduction. Of particular interest are hypervirulent or emerging clones—most notably clonal complex (CC) 17, a lineage strongly associated with neonatal meningitis and LOD and characterized by hypervirulent GBS adhesin (HvgA) and the serine-rich repeat protein Srr2 [8–10], and CC452, an emerging capsular type IV lineage increasingly reported in carriage and invasive diseases that also harbors *srr2* [11–13]. Among the maternal-colonizing isolates, characterization of capsular type, CC/sequence type (ST), virulence factors, and antimicrobial susceptibility profiles is critical for assessing vaccine antigen coverage and interpreting changes following vaccine implementation.

Although several studies have described maternal GBS colonization and related invasive disease in Japan, detailed molecular epidemiological data remain limited [14–18]. To address this gap, we conducted a molecular epidemiological study of 219 GBS isolates recovered from vaginal screening samples of pregnant women receiving antenatal care at a general hospital in Osaka Prefecture, Japan. We characterized capsular type distribution, population structure (CC/ST), major surface-antigen repertoires (pilus islands [PIs], Alp family, Srr1/Srr2, and HvgA), and antimicrobial susceptibility. These findings may complement reports from other regions and provide a pre-vaccine baseline for Japan to inform clinical prevention strategies and future vaccine evaluations.

## 2. Materials and methods

### 2.1. Ethics statement

This study was approved by the ethics committee of Mimihara General Hospital (Approval No. 202403-2-1) and the National Institute of Infectious Diseases (Approval No. 1512). Clinical and laboratory data related to maternal GBS screening were accessed for research purposes between 01/05/2024 and 28/06/2024. The study used anonymized clinical and laboratory data. The requirement for written informed consent was waived by the ethics committees, and informed consent was obtained using an opt-out approach.

### 2.2. Bacterial isolates and identification

Maternal GBS screening samples were collected between 11/09/2021 and 19/09/2023. During this period, GBS screening tests were performed in 1,461 women. The screening test was conducted in accordance with the ASM guideline, Guidelines for the Detection and Identification of Group B *Streptococcus* [19]. Pregnant women at 35–37 weeks of gestation were screened using vaginal-rectal swabs. Immediately after collection, the swabs were transported to the in-house laboratory and culture was initiated without delay. The swabs were first inoculated into Todd–Hewitt broth supplemented with colistin and nalidixic acid (Nikken Bio, Inc., Kyoto, Japan), a selective enrichment broth for GBS, and incubated aerobically at 35 °C overnight. The enrichment broth was then subcultured onto selective agar for GBS (Vi GBS agar; Eiken Chemical Co., Ltd., Tokyo, Japan) and incubated aerobically at 35 °C overnight according to the manufacturer’s instructions. All isolates were identified as *S. agalactiae* using matrix-assisted laser desorption/ionization time-of-flight mass spectrometry (MALDI Biotyper; Bruker Daltonics, Bremen, Germany).

### 2.3. Antimicrobial susceptibility testing and capsular typing

Minimum inhibitory concentrations and inducible clindamycin resistance were determined using the broth microdilution method with commercially prepared Dry Plate Eiken DP44 panels (Eiken Chemical Co., Ltd., Tokyo, Japan), according to the manufacturer’s instructions. Susceptibility breakpoints and interpretation of inducible clindamycin resistance were based on the Clinical and Laboratory Standards Institute document M100, 35th edition [20].

**Fig 1:**
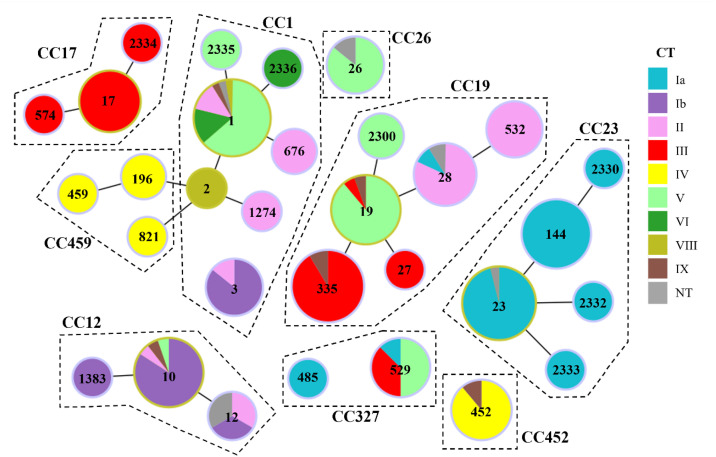
Distribution of sequence types and capsular types on the goeBURST Full MST.

Capsular typing was performed using a GBS latex agglutination test kit, the ImmuLex™ Strep-B Kit (SSI Diagnostica, Hillerød, Denmark), according to the manufacturer’s instructions. The capsular types shown in Tables 1–3 and Fig 1 are based on the latex agglutination results.

**Table 1:** Distribution of sequence types and capsular types by clonal complexes.

CC	ST	Number of isolates by capsular type (%, within each ST)									
		Ia	Ib	II	III	IV	V	VI	VIII	IX	NT
CC1 (n = 46, 21.0%)	ST1 (n = 33, 15.1%)	0	0	4 (12.1)	0	0	21 (63.6)	5 (15.2)	1 (3.0)	1 (3.0)	1 (3.0)
	ST3 (n = 7, 3.2%)	0	6 (85.7)	1 (14.3)	0	0	0	0	0	0	0
	ST676 (n = 2, 0.9%)	0	0	2 (100)	0	0	0	0	0	0	0
	ST2 (n = 1, 0.5%)	0	0	0	0	0	0	0	1 (100)	0	0
	ST1274 (n = 1, 0.5%)	0	0	1 (100)	0	0	0	0	0	0	0
	ST2335 (n = 1, 0.5%)	0	0	0	0	0	1 (100)	0	0	0	0
	ST2336 (n = 1, 0.5%)	0	0	0	0	0	0	1 (100)	0	0	0
CC12 (n = 23, 10.5%)	ST10 (n = 19, 8.7%)	0	16 (84.2)	1 (5.3)	0	0	1 (5.3)	0	0	1 (5.3)	0
	ST12 (n = 3, 1.4%)	0	1 (33.3)	1 (33.3)	0	0	0	0	0	0	1 (33.3)
	ST1383 (n = 1, 0.5%)	0	1 (100)	0	0	0	0	0	0	0	0
CC17 (n = 12, 5.5%)	ST17 (n = 10, 4.6%)	0	0	0	10 (100)	0	0	0	0	0	0
	ST574 (n = 1, 0.5%)	0	0	0	1 (100)	0	0	0	0	0	0
	ST2334 (n = 1, 0.5%)	0	0	0	1 (100)	0	0	0	0	0	0
CC19 (n = 61, 27.9%)	ST335 (n = 23, 10.5%)	0	0	0	21 (91.3)	0	0	0	0	2 (8.7)	0
	ST19 (n = 18, 8.2%)	0	0	0	1 (5.6)	0	16 (88.9)	0	0	1 (5.6)	0
	ST28 (n = 11, 5.0%)	1 (9.1)	0	9 (81.8)	0	0	0	0	0	0	1 (9.1)
	ST532 (n = 6, 2.7%)	0	0	6 (100)	0	0	0	0	0	0	0
	ST2300 (n = 2, 0.9%)	0	0	0	0	0	2 (100)	0	0	0	0
	ST27 (n = 1, 0.5%)	0	0	0	1 (100)	0	0	0	0	0	0
CC23 (n = 48, 21.9%)	ST23 (n = 26, 11.9%)	25 (96.2)	0	0	0	0	0	0	0	0	1 (3.8)
	ST144 (n = 19, 8.7%)	19 (100)	0	0	0	0	0	0	0	0	0
	ST2330 (n = 1, 0.5%)	1 (100)	0	0	0	0	0	0	0	0	0
	ST2332 (n = 1, 0.5%)	1 (100)	0	0	0	0	0	0	0	0	0
	ST2333 (n = 1, 0.5%)	1 (100)	0	0	0	0	0	0	0	0	0
CC26 (n = 7, 3.2%)	ST26 (n = 7, 3.2%)	0	0	0	0	0	6 (85.7)	0	0	0	1 (14.3)
CC327 (n = 9, 4.1%)	ST529 (n = 8, 3.7%)	1 (12.5)	0	0	3 (37.5)	0	4 (50.0)	0	0	0	0
	ST485 (n = 1, 0.5%)	1 (100)	0	0	0	0	0	0	0	0	0
CC452 (n = 9, 4.1%)	ST452 (n = 9, 4.1%)	0	0	0	0	8 (88.9)	0	0	0	1 (11.1)	0
CC459 (n = 4, 1.8%)	ST196 (n = 2, 0.9%)	0	0	0	0	2 (100)	0	0	0	0	0
	ST459 (n = 1, 0.5%)	0	0	0	0	1 (100)	0	0	0	0	0
	ST821 (n = 1, 0.5%)	0	0	0	0	1 (100)	0	0	0	0	0
Total number of isolates (%)		50 (22.8)	24 (11.0)	25 (11.4)	38 (17.4)	12 (5.5)	51 (23.3)	6 (2.7)	2 (0.9)	6 (2.7)	5 (2.3)

CC, clonal complex; ST, sequence type; NT, non-typeable.

**Table 2:** Distribution of pilus island types and major surface protein-associated virulence genes.

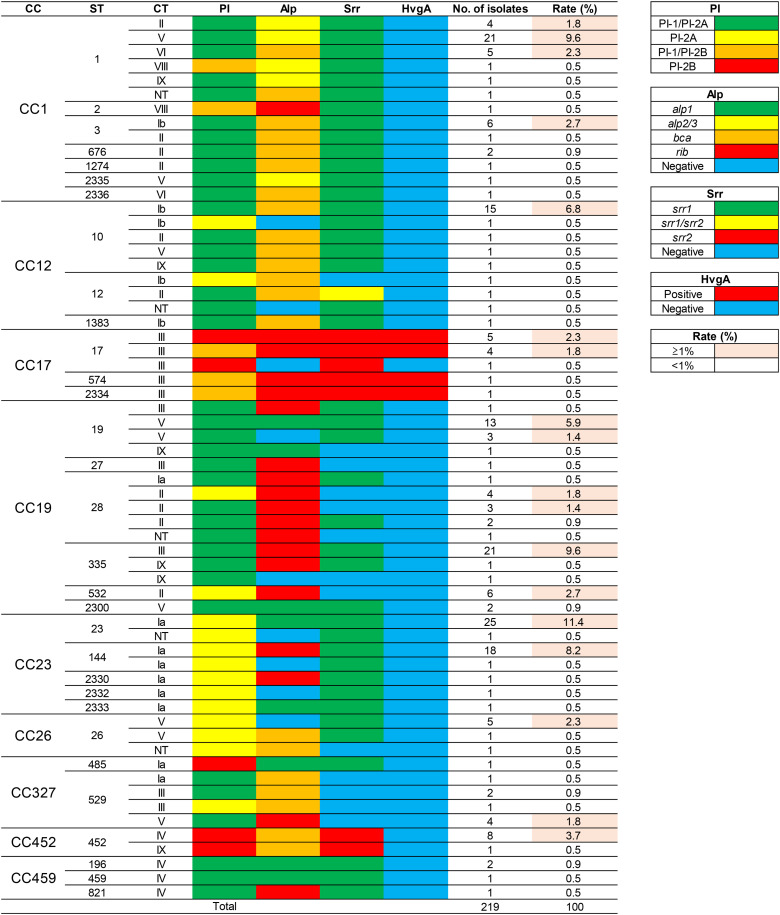

CC, clonal complex; ST, sequence type; CT, capsular type; NT, non-typeable; PI, pilus island; Alp, alpha-like protein (Alp) family; Srr, serine-rich repeat protein; HvgA, hypervirulent GBS adhesin.

**Table 3:** Distribution of antimicrobial susceptibility results for macrolides, clindamycin, minocycline, and levofloxacin by sequence type and capsular type.

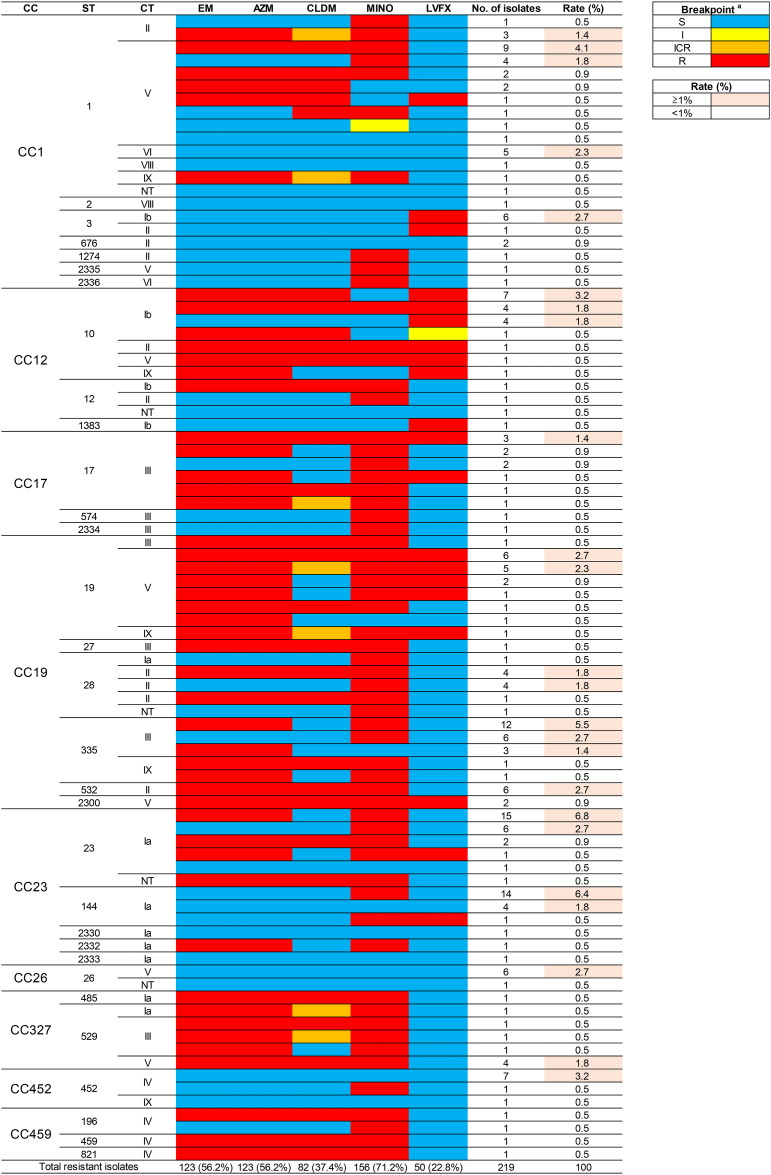

^a^ Penicillin G, cefotaxime, and vancomycin are not shown in Table 3 because all isolates were susceptible to these agents; detailed MIC data are provided in S1 Table.

CC, clonal complex; ST, sequence type; CT, capsular type; NT, non-typeable; EM, erythromycin; AZM, azithromycin; CLDM, clindamycin; MINO, minocycline; LVFX, levofloxacin, S, susceptible; I, intermediate; R, resistant; ICR, inducible clindamycin resistance.

### 2.4. Whole genome sequencing analysis

Genomic DNA was extracted using the QIAamp® DNA Mini Kit (QIAGEN, Hilden, Germany) according to the manufacturer’s instructions. Short-read DNA libraries for Illumina sequencing were prepared using the Enzymatic 5X Whole genome sequencing (WGS) Fragmentation Mix and 5X WGS Ligase Mix (BioStream Corp, Tokyo, Japan) together with an automated library preparation system Biomek i7 Workstation (Beckman Coulter Inc., Brea, CA, USA).

Paired-end 150-bp short-reads were generated using the NovaSeq X Plus platform (Illumina, San Diego, CA, USA). In silico multilocus sequence typing, antimicrobial resistance gene detection, and surface protein detection, including candidate vaccine targets, were performed using an in-house pipeline [21]. This pipeline included in silico capsular typing with GBS-SBG [22]. CCs were identified using the *S. agalactiae* database on the PubMLST.org website [23]. Genetic relationships among the identified STs were analyzed using the goeBURST Full MST algorithm implemented in PHYLOViZ 2.0 [24].

## 3. Results

### 3.1. Distribution of CCs, STs, and capsular types

Among the 1,461 study participants, GBS was detected in 273 women (18.7%). Of these, 219 isolates were preserved and subjected to subsequent analysis.

The distribution of CCs, STs, and capsular types among the 219 GBS isolates is summarized in Fig 1, and detailed data are presented in Table 1. The isolates were assigned to 31 STs, including seven novel STs, and grouped into nine CCs. CC19 was the most frequent lineage (n = 61, 27.9%), followed by CC23 (n = 48, 21.9%) and CC1 (n = 46, 21.0%). The hypervirulent lineage CC17 accounted for 12 isolates (5.5%) and ranked fifth overall.

The capsular type distribution was as follows: Ia, 50 (22.8%); Ib, 24 (11.0%); II, 25 (11.4%); III, 38 (17.4%); IV, 12 (5.5%); V, 51 (23.3%); VI, 6 (2.7%); VIII, 2 (0.9%); IX, 6 (2.7%); and non-typeable, 5 (2.3%). Among the 219 isolates, 14 (6.4%) showed discordant capsular typing results between latex agglutination and WGS-based analysis. These included six isolates identified as capsular type IX and five non-typeable isolates by latex agglutination, all of which were assigned to other capsular types by WGS-based analysis (S1 Table). The dominant CCs within each capsular type were CC23 for Ia, CC12 for Ib, CC19 and CC1 for II, CC17 and CC19 for III, CC452 for IV, and CC1 and CC19 for V. CC1 was represented across all capsular types except Ia, III, and IV.

Multilocus sequence typing identified 31 sequence types (STs) represented by nodes. The size of each node was proportional to the number of isolates on a logarithmic scale. The goeBURST Full MST algorithm was used to visualize the genetic relationships among STs based on their allelic profiles. Node colors distinguish sub-group founders (dark green) from other nodes (light blue). Colored sectors within each ST node indicate the distribution of capsular types (Ia, Ib, II, III, IV, V, VI, VIII, IX, and non-typeable [NT]). Links between nodes represent single-locus variants. Dashed lines delineate the nine identified clonal complexes (CCs).

### 3.2. Distribution of PI types and major surface protein-associated virulence genes

Sequencing statistics are shown in S1 Table. The average coverage (±standard deviation) was 412 (±135). Draft genome data are available under accession number PRJDB39678.

PI profiles were distributed as follows; PI-1/PI-2A (n = 127, 58.0%), PI-2A alone (n = 68, 31.1%), PI-2B alone (n = 16, 7.3%), and PI-1/PI-2B (n = 8, 3.7%). Within the Alp family genes, *rib* was the most common (n = 77, 35.2%), followed by *bca* (n = 53, 24.2%), *alp1* (n = 46, 21.0%), and *alp2/3* (n = 28, 12.8%); 15 isolates (6.8%) lacked all four of these Alp genes (Table 2).

Regarding serine-rich repeat adhesins, *srr1* was detected in 170 isolates (77.6%) and *srr2* in 21 isolates (9.6%). *srr2*-positive isolates were almost exclusively confined to CC17/capsular type III and CC452/ capsular type IV. The hypervirulence-associated adhesin gene *hvgA* was present in 11 isolates (5.0%), all belonging to CC17/ capsular type III (Table 2).

### 3.3. Antimicrobial susceptibility and resistance determinants

Antimicrobial susceptibility results and detailed resistance profiles are presented in Table 3 and S1 Table. All isolates were susceptible to β-lactams. In contrast, macrolide resistance was common: 123 isolates (56.2%) were resistant to erythromycin, and all erythromycin resistant isolates were also resistant to azithromycin. Among these isolates, the most frequently detected macrolide resistance determinant was *ermB* (64 isolates, 52.9%), followed by co-detection of *mefA* and *msrD* (32 isolates, 26.4%) and *ermA* (27 isolates, 22.3%). Macrolide resistance was frequent in ST1, ST10, ST17, ST23, CC19 and CC327.

Clindamycin resistance was observed in 82 isolates (37.4%), including 13 isolates exhibiting inducible resistance. Among isolates characterized as clindamycin-susceptible or clindamycin-intermediate, 8.7% displayed inducible resistance on confirmatory testing.

Minocycline resistance was observed in 156 isolates (71.2%). Among these isolates, *tetM* was the most frequently detected tetracycline resistance gene (122 isolates, 78.2%), followed by *tetO* (34 isolates, 21.8%). Among the ten capsular type III/ST17 isolates, six exhibited a characteristic profile comprising: *ermB* positivity, *tetO* positivity, PI-2B positivity, and absence of PI-1. Minocycline was included for epidemiological purposes to characterize tetracycline-class resistance phenotypes associated with resistance determinants such as *tetM* and *tetO*, rather than as a therapeutic option during pregnancy.

Levofloxacin resistance was observed in 50 isolates (22.8%) and was enriched in ST3, ST10, and ST19, as well as in capsular types Ib and V. Most levofloxacin-resistant isolates harbored two quinolone resistance-associated amino acid substitutions; GyrA S81L together with either ParC S79F or ParC S79Y.

## 4. Discussion

In this study, we characterized 219 colonizing GBS isolates from pregnant women in Osaka Prefecture with respect to CC/ST and capsular type distribution, virulence factors, and antimicrobial susceptibility. Detailed assessment of virulence factors provided insights into the characteristics of maternal colonizing strains and their potential to cause invasive diseases.

The CC lineage distribution observed in our isolates, predominantly CC19, CC23, and CC1, was consistent with findings from other maternal carriage studies in Japan. For example, Kawaguchiya et al. reported that CC19 (29%), CC23 (25%), and CC1 (17%) were the major lineages among pregnant women in Hokkaido [16]. Hypervirulent CC17 accounted for 5.5% of the isolates in our study, which is lower than the carriage rate of approximately 10% reported in previous studies [16,25]. Capsular type III accounted for 17.4% of all isolates, despite the relatively low frequency of CC17. This discrepancy is largely explained by the high prevalence of capsular type III/CC19 (ST335) isolates in our collection, a distribution pattern consistent with a previous report [16]. In contrast, invasive disease surveillance studies have consistently shown that CC17 predominates among isolates causing neonatal meningitis and LOD. For example, Takeuchi et al. identified CC17 as the leading clone among invasive cases in Chiba Prefecture [15]. In Japan, a recent nationwide surveillance study reported that 21.5% of infants with GBS meningitis develop neurodevelopmental sequelae [14]. Because capsular type III/CC17 accounts for the majority of neonatal GBS meningitis cases worldwide, these findings suggest that the hypervirulent CC17 clone is a major contributor to meningitis-associated neurological sequelae. Taken together, these observations indicate that although CC17 is carried by only a small proportion of pregnant women, it contributes disproportionately to neonatal diseases.

Recent epidemiological and genomic studies from other regions have highlighted both shared and region-specific features of GBS epidemiology. In Argentina, a national multicenter genomic study of human GBS isolates collected during 2014–2015 from invasive disease, urinary tract infections, and maternal colonization showed that capsular type Ia/CC23 and capsular type Ib/CC12 were the most prevalent lineages regardless of isolation source, whereas capsular type III/CC19 exhibited a high density of prophages, virulence factors, and antimicrobial resistance determinants [26]. In Brazil, recent genomic epidemiological analysis of GBS isolates from colonized pregnant women identified the neonatal disease-associated CC23 lineage as the predominant lineage [27]. In coastal Kenya, WGS analysis of maternal colonizing and neonatal disease isolates demonstrated a maternal colonization prevalence of approximately 12% and a substantial contribution of CC17 to both maternal colonization and neonatal disease [28]. In Western Australia, WGS of perinatal isolates collected from pregnant women during 2015–2017 and neonatal invasive isolates collected during 2012–2014 showed that the major clonal complexes were CC1, CC12, CC17, CC19, and CC23 [29]. These findings suggest that the major colonizing lineages observed in Japan partially overlap with global GBS populations, although their relative prevalence differs substantially among regions.

The detection of ST452, although limited in number, warrants special attention. This lineage, associated with capsular type IV and *srr2* positivity, is internationally recognized as an emerging invasive clone. Campisi et al. demonstrated that capsular type IV/ST452 likely arose through large genomic recombination events between CC23 and the hypervirulent CC17 lineage [30]. Teatero et al. also identified ST452 among invasive capsular type IV GBS isolates in Toronto, Canada [31]. In Japan, Kasai et al. demonstrated that most capsular type IV pediatric invasive isolates detected since 2019 belonged to CC452, including ST452 and single-locus variants of ST452 [13]. Recent genomic data from Argentina further identified capsular type Ia/CC452 among human-associated GBS lineages, indicating that CC452-related lineages are also present in South America [26]. Together with the identification of *srr2*-positive CC452 isolates in the present study, these observations support the inclusion of ST452/CC452-related lineages in ongoing maternal carriage and invasive disease surveillance.

Regarding PI distribution, PI-1/PI-2A was the predominant profile among our isolates. This finding is consistent with previous studies showing that PI-1/PI-2A is commonly observed among maternal colonizing isolates, whereas PI-2B is closely associated with capsular type III/ST17 invasive isolates [32,33]. The low frequency of PI-1/PI-2B (3.7%) in our study likely reflects the limited representation of hypervirulent CC17 lineages in maternal carriage. Notably, 60% of the capsular type III/ST17 isolates identified in this study were *ermB*- and *tetO*-positive and carried PI-2B alone. Isolates with these characteristics have been reported globally [34–36] and this clone has also been detected at high frequencies among pediatric invasive GBS isolates in Japan [13]. These findings suggest that maternal carriage may contribute to the maintenance and dissemination of this GBS clone.

Among the Alp family genes, *rib* was the most prevalent, consistent with findings from international studies showing that rib predominates in capsular type III and CC17 isolates [1]. However, in our collection, rib was also frequently observed in CC19 and CC23, indicating that this antigen is not restricted to invasive clones in Japan. Conversely, *alp2/3* was less frequent (12.8%), consistent with its limited distribution among CC1 lineages reported in other regions [37]. These observations suggest that colonizing GBS strains in Japan share conserved surface protein repertoires with strains reported globally, thereby providing useful information for vaccine antigen selection.

The distribution of Srr adhesins also provides insight into potential virulence. The predominance of *srr1* (77.6%) and the limited presence of *srr2* (9.6%) were consistent with global observations that *srr1* is widespread among colonizing isolates, whereas *srr2* is strongly associated with hypervirulent CC17 lineages [9]. Mechanistic studies have shown that Srr2 mediates stronger binding to fibrinogen than Srr1 and also interacts with fibronectin, thereby enhancing adhesion to host ligands and cervicovaginal epithelial cells [38,39]. In our study, *srr2* was confined to CC17 and CC452, suggesting that CC452 may share adhesive or invasive potential with the prototypical hypervirulent CC17 lineage. The detection of *srr2*-positive capsular type IV/CC452 colonizing isolates, which has rarely been reported in Japan, further supports the need for continued monitoring of this lineage.

The antimicrobial resistance patterns observed in our isolates were broadly consistent with those reported in a recent Japanese study of GBS isolates from pregnant women, in which susceptibility to β-lactams was preserved, whereas resistance to macrolides, tetracycline-class agents, and levofloxacin was observed [16]. All isolates in our study remained susceptible to β-lactams and vancomycin. In contrast, resistance to macrolides and tetracycline-class agents was common. Macrolide resistance was mainly associated with *ermB*, co-detection of *mefA* and *msrD*, and *ermA*, whereas tetracycline-class resistance among minocycline-resistant isolates was predominantly associated with *tetM* and *tetO*. This pattern is broadly consistent with recent genomic studies of GBS resistance determinants, including studies of maternal colonizing isolates from Brazil and pediatric invasive isolates from Japan [13,27]. Although minocycline is not used therapeutically during pregnancy, its susceptibility profile was useful for epidemiological characterization of resistant clones. Levofloxacin resistance was enriched in ST3, ST10, and ST19 and was mainly associated with canonical quinolone resistance-determining region (QRDR) mutations, similar to findings reported among pediatric invasive GBS isolates in Japan [13]. Taken together, the coexistence of colonization-associated virulence profiles, such as PI-1/PI-2A and *srr1*, with multiple antimicrobial resistance traits suggests that even non-hypervirulent maternal carriage strains may serve as reservoirs of antimicrobial-resistant GBS.

In our isolate collection, the predicted vaccine coverage exceeded 90% for both the hexavalent capsular polysaccharide conjugate vaccine targeting capsular types Ia, Ib, II, III, IV, and V (GBS6) and protein-based vaccines directed against Alp family antigens (GBS-NN/NN2). These findings suggest that current vaccine designs broadly target the major features of typical invasive lineages from a clinical perspective [7]. Nevertheless, a subset of maternal colonizing strains may remain outside the expected coverage spectrum, and shifts in the distribution of epidemic clones following vaccine implementation are possible. Continued epidemiological surveillance is therefore warranted to identify potential gaps in coverage and detect clonal replacement.

This study has several limitations. First, it was restricted to isolates collected in Osaka Prefecture, and the sample size may not fully represent nationwide carriage patterns. Second, functional assays to confirm gene expression were not performed, and the associations between virulence factors and invasiveness therefore remain inferential. Nevertheless, the detailed mapping of virulence loci in maternal carriage isolates provides a valuable pre-vaccine baseline and a molecular framework for ongoing national surveillance.

In conclusion, our analysis demonstrated that GBS colonizing pregnant women in Osaka was predominantly composed of CC19, CC23, and CC1, which are typical colonization-associated lineages. Although CC17 remained infrequent, it carried all canonical hypervirulence markers (*hvgA*, *srr2*, PI-1/PI-2B). The identification of *srr2*-positive CC452 (capsular type IV) suggests the emergence of a potentially important lineage. As vaccine introduction approaches, continued monitoring of CC/ST distribution, capsular types, virulence factors, and antimicrobial susceptibility will be essential to anticipate epidemiological shifts and sustain progress in the prevention of early- and late-onset GBS disease.

## Supporting information

S1 Table Molecular epidemiological characteristics of Group B *Streptococcus* carried by pregnant women at a general hospital in Osaka prefecture, Japan. (XLSX)

## Abbreviations

GBS: Group B *Streptococcus*
CC: clonal complex
ST: sequence type
EOD: early-onset disease
LOD: late-onset disease
Alp: alpha-like protein
HvgA: hypervirulent GBS adhesin
PI: pilus island.

## References

[1] Seale AC, Bianchi-Jassir F, Russell NJ, Kohli-Lynch M, Tann CJ, Hall J, et al. Estimates of the Burden of Group B Streptococcal Disease Worldwide for Pregnant Women, Stillbirths, and Children. Clin Infect Dis. 2017;65(suppl_2):S200–19. doi: 10.1093/cid/cix664 29117332 PMC5849940

[2] Russell NJ, Seale AC, O’Driscoll M, O’Sullivan C, Bianchi-Jassir F, Gonzalez-Guarin J, et al. Maternal Colonization With Group B *Streptococcus* and Serotype Distribution Worldwide: Systematic Review and Meta-analyses. Clin Infect Dis. 2017;65(suppl_2):S100–11. doi: 10.1093/cid/cix658 29117327 PMC5848259

[3] Shabayek S, Spellerberg B. Group B streptococcal colonization, molecular characteristics, and epidemiology. Front Microbiol. 2018;9:437. doi: 10.3389/fmicb.2018.00437 29593684 PMC5861770

[4] Absalon J, Segall N, Block SL, Center KJ, Scully IL, Giardina PC, et al. Safety and immunogenicity of a novel hexavalent group B streptococcus conjugate vaccine in healthy, non-pregnant adults: a phase 1/2, randomised, placebo-controlled, observer-blinded, dose-escalation trial. Lancet Infect Dis. 2021;21(2):263–74. doi: 10.1016/S1473-3099(20)30478-3 32891191 PMC9760110

[5] Madhi SA, Anderson AS, Absalon J, Radley D, Simon R, Jongihlati B, et al. Potential for Maternally Administered Vaccine for Infant Group B Streptococcus. N Engl J Med. 2023;389(3):215–27. doi: 10.1056/NEJMoa2116045 37467497

[6] Gonzalez-Miro M, Pawlowski A, Lehtonen J, Cao D, Larsson S, Darsley M, et al. Safety and immunogenicity of the group B *streptococcus* vaccine AlpN in a placebo-controlled double-blind phase 1 trial. iScience. 2023;26(3):106261. doi: 10.1016/j.isci.2023.106261 36915681 PMC10005905

[7] Pena JMS, Lannes-Costa PS, Nagao PE. Vaccines for *Streptococcus agalactiae*: current status and future perspectives. Front Immunol. 2024;15:1430901. doi: 10.3389/fimmu.2024.1430901 38947337 PMC11211565

[8] Tazi A, Disson O, Bellais S, Bouaboud A, Dmytruk N, Dramsi S, et al. The surface protein HvgA mediates group B streptococcus hypervirulence and meningeal tropism in neonates. J Exp Med. 2010;207(11):2313–22. doi: 10.1084/jem.20092594 20956545 PMC2964583

[9] Six A, Bellais S, Bouaboud A, Fouet A, Gabriel C, Tazi A, et al. Srr2, a multifaceted adhesin expressed by ST-17 hypervirulent Group B *Streptococcus* involved in binding to both fibrinogen and plasminogen. Mol Microbiol. 2015;97(6):1209–22. doi: 10.1111/mmi.13097 26094503

[10] Liu Y, Liu J. Group B Streptococcus: Virulence Factors and Pathogenic Mechanism. Microorganisms. 2022;10:2483. doi: 10.3390/microorganisms10122483 36557736 PMC9784991

[11] Diedrick MJ, Flores AE, Hillier SL, Creti R, Ferrieri P. Clonal analysis of colonizing group B *Streptococcus*, serotype IV, an emerging pathogen in the United States. J Clin Microbiol. 2010;48:3100–4. doi: 10.1128/jcm.00277-10 20610684 PMC2937746

[12] Creti R, Imperi M, Khan UB, Berardi A, Recchia S, Alfarone G, et al. Emergence of High-Level Gentamicin Resistance in *Streptococcus agalactiae* Hypervirulent Serotype IV ST1010 (CC452) Strains by Acquisition of a Novel Integrative and Conjugative Element. Antibiotics (Basel). 2024;13(6):491. doi: 10.3390/antibiotics13060491 38927158 PMC11201010

[13] Kasai M, Nakano S, Koide S, Otake S, Shibata M, Ishida-Kuroki K, et al. Prevalence of Candidate Vaccine Targets and Genomic Features of Pediatric Invasive Streptococcus Agalactiae in Japan. J Infect Dis. 2026;233(1):e11–21. doi: 10.1093/infdis/jiaf491 41065369 PMC12811887

[14] Shibata M, Matsubara K, Matsunami K, Miyairi I, Kasai M, Kai M, et al. Epidemiology of group B streptococcal disease in infants younger than 1 year in Japan: a nationwide surveillance study 2016–2020. Eur J Clin Microbiol Infect Dis. 2022;41:559–71. doi: 10.1007/s10096-021-04396-y 35048277

[15] Takeuchi N, Chang B, Takeshita K, Naito S, Takahashi Y, Hishiki H, et al. Epidemiology and bacterial characteristics of invasive group B streptococcus disease: a population-based study in Japan in 2010-2020. Epidemiol Infect. 2022;150:e184. doi: 10.1017/S0950268822001534 36408537 PMC9987023

[16] Kawaguchiya M, Urushibara N, Aung MS, Shimada S, Nakamura M, Ito M, et al. Molecular characterization and antimicrobial resistance of *Streptococcus agalactiae* isolated from pregnant women in Japan, 2017–2021. IJID Reg. 2022;4:143–5. doi: 10.1016/j.ijregi.2022.07.002 35923645 PMC9340534

[17] Morozumi M, Wajima T, Kuwata Y, Chiba N, Sunaoshi K, Sugita K, et al. Associations between capsular serotype, multilocus sequence type, and macrolide resistance in *Streptococcus agalactiae* isolates from Japanese infants with invasive infections. Epidemiol Infect. 2014;142(4):812–9. doi: 10.1017/S0950268813001647 23866831 PMC9151080

[18] Chang B, Wada A, Hosoya M, Oishi T, Ishiwada N, Oda M, et al. Characteristics of group B *Streptococcus* isolated from infants with invasive infections: a population-based study in Japan. Jpn J Infect Dis. 2014;67(5):356–60. doi: 10.7883/yoken.67.356 25241685

[19] Filkins L, Hauser J, Robinson-Dunn B, Tibbetts R, Boyanton B, Revell P. Guidelines for the Detection and Identification of Group B *Streptococcus*. Washington (DC): American Society for Microbiology; 2021. 10.1128/JCM.01230-20 PMC7771461 33115849

[20] Clinical and Laboratory Standards Institute. Performance Standards for Antimicrobial Susceptibility Testing. 35th ed. CLSI supplement M100. Clinical and Laboratory Standards Institute; 2025.

[21] Nakano S, Koide S, Hosaka Y, Hasegawa Y, Ishida-Kuroki K, Kawakami S, et al. Enrichment culture evaluation and characterization of *Streptococcus agalactiae* among pregnant women in Japan. J Med Microbiol. 2024;73(7). doi: 10.1099/jmm.0.001849 38985141

[22] Tiruvayipati S, Tang WY, Barkham TMS, Chen SL. GBS-SBG - GBS Serotyping by Genome Sequencing. Microb Genom. 2021;7(12):000688. doi: 10.1099/mgen.0.000688 34895403 PMC9842102

[23] Jolley KA, Bray JE, Maiden MCJ. Open-access bacterial population genomics: BIGSdb software, the PubMLST.org website and their applications. Wellcome Open Res. 2018;3:124. doi: 10.12688/wellcomeopenres.14826.1 30345391 PMC6192448

[24] Nascimento M, Sousa A, Ramirez M, Francisco AP, Carriço JA, Vaz C. PHYLOViZ 2.0: providing scalable data integration and visualization for multiple phylogenetic inference methods. Bioinformatics. 2017;33(1):128–9. doi: 10.1093/bioinformatics/btw582 27605102

[25] Jamrozy D, Gopal Rao G, Feltwell T, Lamagni T, Khanna P, Efstratiou A, et al. Population genetics of group B *Streptococcus* from maternal carriage in an ethnically diverse community in London. Front Microbiol. 2023;14:1185753. doi: 10.3389/fmicb.2023.1185753 37275158 PMC10233156

[26] Kovacec V, Di Gregorio S, Pajon M, Khan UB, Poklepovich T, Campos J, et al. Genomic characterization of group B Streptococcus from Argentina: insights into prophage diversity, virulence factors and antibiotic resistance genes. Microb Genom. 2025;11(4):001399. doi: 10.1099/mgen.0.001399 40266661 PMC12046356

[27] Oliveira L, Costa N, Simões L, Marinho P, Fracalanzza S, Teixeira L. Group B Streptococcus genomic epidemiology indicates that the neonatal disease-associated clonal complex 23 is the major lineage found colonizing pregnant women in Brazil. Int J Infect Dis. 2025;152:107608. doi: 10.1016/j.ijid.2024.107608

[28] Seale AC, Koech AC, Sheppard AE, Barsosio HC, Langat J, Anyango E, et al. Maternal colonization with Streptococcus agalactiae and associated stillbirth and neonatal disease in coastal Kenya. Nat Microbiol. 2016;1(7):16067. doi: 10.1038/nmicrobiol.2016.67 27572968 PMC4936517

[29] Furfaro LL, Chang BJ, Kahler CM, Payne MS. Genomic characterisation of perinatal Western Australian Streptococcus agalactiae isolates. PLoS One. 2019;14(10):e0223256. doi: 10.1371/journal.pone.0223256 31577825 PMC6774530

[30] Campisi E, Rinaudo CD, Donati C, Barucco M, Torricelli G, Edwards MS, et al. Serotype IV *Streptococcus agalactiae* ST-452 has arisen from large genomic recombination events between CC23 and the hypervirulent CC17 lineages. Sci Rep. 2016;6:29799. doi: 10.1038/srep29799 27411639 PMC4944191

[31] Teatero S, McGeer A, Li A, Gomes J, Seah C, Demczuk W, et al. Population structure and antimicrobial resistance of invasive serotype IV group B *Streptococcus*, Toronto, Ontario, Canada. Emerg Infect Dis. 2015;21:585–91. doi: 10.3201/eid2014.140759 25811284 PMC4378482

[32] Madzivhandila M, Adrian PV, Cutland CL, Kuwanda L, Madhi SA, The PoPS Trial Team. Distribution of pilus islands of group B streptococcus associated with maternal colonization and invasive disease in South Africa. J Med Microbiol. 2013;62(Pt 2):249–53. doi: 10.1099/jmm.0.052951-0 23065545

[33] Choi JH, Kim TH, Kim ET, Kim YR, Lee H. Molecular epidemiology and virulence factors of group B Streptococcus in South Korea according to the invasiveness. BMC Infect Dis. 2024;24(1):740. doi: 10.1186/s12879-024-09625-1 39060964 PMC11282841

[34] Teatero S, Ramoutar E, McGeer A, Li A, Melano RG, Wasserscheid J, et al. Clonal Complex 17 Group B *Streptococcus* strains causing invasive disease in neonates and adults originate from the same genetic pool. Sci Rep. 2016;6:20047. doi: 10.1038/srep20047 26843175 PMC4740736

[35] Campisi E, Rosini R, Ji W, Guidotti S, Rojas-López M, Geng G, et al. Genomic Analysis Reveals Multi-Drug Resistance Clusters in Group B *Streptococcus* CC17 Hypervirulent Isolates Causing Neonatal Invasive Disease in Southern Mainland China. Front Microbiol. 2016;7:1265. doi: 10.3389/fmicb.2016.01265 27574519 PMC4983569

[36] Plainvert C, Hays C, Touak G, Joubrel-Guyot C, Dmytruk N, Frigo A. Multidrug-Resistant Hypervirulent Group B *Streptococcus* in Neonatal Invasive Infections, France, 2007–2019. Emerg Infect Dis. 2020;26:2721–4. doi: 10.3201/eid2611.201669 33079049 PMC7588536

[37] Mohammadi A, Amini C, Bagheri P, Salehi Z, Goudarzi M. Unveiling the genetic landscape of *Streptococcus agalactiae* bacteremia: emergence of hypervirulent CC1 strains and new CC283 strains in Tehran, Iran. BMC Microbiol. 2024;24(1):365. doi: 10.1186/s12866-024-03521-z 39342084 PMC11438095

[38] Seo HS, Minasov G, Seepersaud R, Doran KS, Dubrovska I, Shuvalova L, et al. Characterization of fibrinogen binding by glycoproteins Srr1 and Srr2 of *Streptococcus agalactiae*. J Biol Chem. 2013;288(50):35982–96. doi: 10.1074/jbc.M113.513358 24165132 PMC3861647

[39] Pellegrini A, Motta C, Bellan Menegussi E, Pierangelini A, Viglio S, Coppolino F, et al. The serine-rich repeat glycoprotein Srr2 mediates Streptococcus agalactiae interaction with host fibronectin. BMC Microbiol. 2024;24(1):221. doi: 10.1186/s12866-024-03374-6 38909237 PMC11193222

